# CB_1_ Cannabinoid Receptors Stimulate Gβγ-GRK2-Mediated FAK Phosphorylation at Tyrosine 925 to Regulate ERK Activation Involving Neuronal Focal Adhesions

**DOI:** 10.3389/fncel.2020.00176

**Published:** 2020-06-23

**Authors:** George D. Dalton, Skyla T. Carney, Jamie D. Marshburn, Derek C. Norford, Allyn C. Howlett

**Affiliations:** ^1^Department of Physiology and Pharmacology, Wake Forest University School of Medicine, Winston-Salem, NC, United States; ^2^Department of Biological and Biomedical Sciences, Julius L. Chambers Biomedical and Biotechnology Research Institute, North Carolina Central University, Durham, NC, United States

**Keywords:** CB_1_, FAK, ERK, GRK2, Gβγ, Grb2

## Abstract

CB_1_ cannabinoid receptors (CB_1_) are abundantly expressed in the nervous system where they regulate focal adhesion kinase (FAK) and the mitogen-activated protein kinases (MAPK) extracellular signal-regulated kinase 1 and 2 (ERK1/2). However, the role of CB_1_-stimulated FAK 925 tyrosine phosphorylation (Tyr-P) in regulating ERK1/2 activation remains undefined. Here, immunoblotting analyses using antibodies against FAK phospho-Tyr 925 and ERK2 phospho-Tyr 204 demonstrated CB_1_-stimulated FAK 925 Tyr-P and ERK2 204 Tyr-P (0–5 min) which was followed by a decline in Tyr-P (5–20 min). CB_1_ stimulated FAK-Grb2 association and Ras-mediated ERK2 activation. The FAK inhibitors Y11 and PF 573228 abolished FAK 925 Tyr-P and partially inhibited ERK2 204 Tyr-P. FAK 925 Tyr-P and ERK2 204 Tyr-P were adhesion-dependent, required an intact actin cytoskeleton, and were mediated by integrins, Flk-1 vascular endothelial growth factor receptors, and epidermal growth factor receptors. FAK 925 Tyr-P and ERK2 204 Tyr-P were blocked by the Gβγ inhibitor gallein, a GRK2 inhibitor, and GRK2 siRNA silencing, suggesting Gβγ and GRK2 participate in FAK-mediated ERK2 activation. Together, these studies indicate FAK 925 Tyr-P occurs concurrently with CB_1_-stimulated ERK2 activation and requires the actin cytoskeleton and G_i/o_βγ-GRK2-mediated cross-talk between CB_1_, integrins, and receptor tyrosine kinases (RTKs).

## Introduction

Endocannabinoid signaling in neuronal cells includes a rapid activation of focal adhesion kinase (FAK; Derkinderen et al., 2003; Dalton et al., 2013). FAK is a highly conserved non-receptor protein Tyr kinase that functions as a signal-transducing scaffold protein that regulates multiple cellular functions including proliferation, apoptosis, organization of the actin cytoskeleton, migration, and adhesion (Peng and Guan, 2011). FAK is typically located in focal adhesions where integrin clusters link proteins of the extracellular matrix (ECM) to the actin cytoskeleton. FAK binds to the adaptor protein paxillin to dock with components of the actin cytoskeleton at focal adhesion sites (Parsons, 2003). FAK activation is regulated by Tyr-P and occurs in response to integrin engagement, as well as RTK and G protein-coupled receptor (GPCR) stimulation (Parsons, 2003; Peng and Guan, 2011; Dalton et al., 2013). Tyr 397 is the FAK autophosphorylation site and phosphorylation at this site triggers FAK initial activation. Src family kinases (Src) bind to FAK phospho-Tyr 397 and phosphorylate FAK on additional Tyr residues (Tyr 407, Tyr 576/577, Tyr 861, Tyr 925) that mediate specific FAK functions such as FAK maximal activation and FAK-mediated activation of Ras signal transduction (Schlaepfer et al., 1994; Calalb et al., 1995, 1996; Schlaepfer and Hunter, 1996).

In both neuronal and non-neuronal cell types, modulation of CB_1_ activity induced changes in actin/cytoskeletal reorganization that impacted cell motility, morphology, proliferation, and hormone/neurotransmitter release (Grimaldi et al., 2006; Pisanti et al., 2011; Malenczyk et al., 2013; Roland et al., 2014; Njoo et al., 2015; Wang et al., 2016; Hohmann et al., 2019). FAK plays a key role in all of these cellular processes and studies suggest CB_1_ regulates FAK activity *via* integrin/RTK activation, Src activation, and Protein Kinase A inhibition (Derkinderen et al., 1996, 2001; Karanian et al., 2005; Dalton et al., 2013). Previous investigations in our laboratory of FAK Tyr-P in a neuronal cell model demonstrated that cannabinoid agonists could promote FAK Tyr 397 phosphorylation as well as phosphorylation at the Tyr 576-577 loci (Dalton et al., 2013). Following FAK 397 Tyr-P, Src-mediated FAK 925 Tyr-P creates a binding site for the adaptor protein Grb2 which in many cell types links FAK to activation of the MAPKs ERK1/2 *via* the Grb2/Sos1/Ras pathway (Schlaepfer et al., 1994; Schlaepfer and Hunter, 1996; Mitra et al., 2006; Cheung et al., 2008). As a result of this signaling pathway, FAK 925 Tyr-P could regulate ERK1/2-mediated cell adhesion, migration, survival, and proliferation (Roskoski, 2012). Studies have shown that CB_1_ regulates ERK1/2 phosphorylation/activation *via* several mechanisms that include G_i/o_ protein activation (Galve-Roperh et al., 2002; Davis et al., 2003; Dalton and Howlett, 2012), RTK transactivation (Dalton and Howlett, 2012), and Src activation (Derkinderen et al., 2003; Dalton and Howlett, 2012) under various stimuli and conditions. Given the importance of FAK and ERK1/2 in the endocannabinoid system’s role in neuronal migration and neuritogenesis, it is important to determine whether CB_1_-mediated FAK 925 Tyr-P is required for efficient ERK1/2 pathway activation in neuronal focal adhesions (Cox et al., 2006; Harkany et al., 2008; Samuels et al., 2009; Zorina et al., 2010; Shum et al., 2020).

## Materials and Methods

### Materials

Reagents were purchased from Sigma Chemical Company (St. Louis, MO, USA), unless otherwise stated. SR141716A [*N*-(piperidin-1-yl)-5-(4-chlorophenyl)-1-(2,4-dichlorophenyl)-4-methyl-*H*-pyrazole-3-carboxamide] was provided by the National Institute of Drug Abuse drug supply program. WIN55212-2, farnesylthiosalicylic acid (FTA), and tetrahydrolipstatin (THL, Orlistat) were from Cayman Chemical (Ann Arbor, MI, USA). Acrylamide, *N,N,N′,N′*-tetramethylethylene diamine (TEMED), and sodium dodecyl sulfate (SDS) were from BioRad Laboratories, Inc. (Hercules, CA, USA). GRK2 siRNA (mouse), control siRNA-A, anti-p-FAK (Tyr 397, 2D11), anti-p-FAK (Tyr 925), anti-FAK (H-1), anti-FAK (A-17), anti-ERK2 (K-23), anti-p-ERK (Tyr 204, E-4), anti-Grb2 (C-23), anti-GRK2 (C-9), AG 1478, and anti-β-actin (ACTBD11B7) were from Santa Cruz Biotechnology (Santa Cruz, CA, USA). Y11 and PF 573228 were from Tocris Bioscience (Bristol, UK). The GRK2 inhibitor (methyl 5-[2-(5-nitro-2-furyl)vinyl]-2-furoate) was purchased from EMD Millipore (Billerica, MA, USA) and is a selective β-adrenergic receptor kinase 1 (βARK1) inhibitor that inhibits βARK1 in a concentration-dependent manner (Iino et al., 2002). Gallein, SU 5416, and cytochalasin D were from EMD Millipore. Jasplakinolide, latrunculin A, Alexa Fluor 488 phalloidin, Prolong Antifade reagent, and Texas Red DNase I were from Molecular Probes (Eugene, OR, USA). RGDS peptide was from Abbiotec, LLC (San Diego, CA, USA). Odyssey Blocking buffer, nitrocellulose membranes, IRDye 800CW goat anti-rabbit secondary antibody, and IRDye 680CW goat anti-mouse secondary antibody were from LI-COR Biosciences (Lincoln, NE, USA). BD Falcon 6-well multiwell plates were from VWR International (Suwanee, GA, USA).

### Cell Culture

N18TG2 neuroblastoma cells (passage numbers 25–50) were maintained at 37°C under a 5% CO_2_ atmosphere in Dulbecco’s Modified Eagle’s Medium (DMEM): Ham’s F-12 (1:1) complete with GlutaMax, sodium bicarbonate, and pyridoxine–HCl, supplemented with penicillin (100 units/ml) and streptomycin (100 μg/ml; Gibco Life Technologies, Gaithersburg, MD, USA) and 10% heat-inactivated bovine serum (JRH Biosciences, Lenexa, KS, USA). An aliquot of cannabinoid drug stocks (stored at −20°C as 10 mM solutions in ethanol) or ethanol (control) was air-dried under sterile conditions in trimethylsilyl-coated glass test tubes and taken up in 100 volumes of 5 mg/ml fatty acid-free bovine serum albumin (BSA) and serially diluted before being added to cells. Where indicated, N18TG2 cells were pretreated with receptor antagonists or other inhibitors before the addition of CB_1_ agonists. Pertussis toxin (List Biological Laboratories, Campbell, CA, USA) was added to cells (100 ng/ml) for 16–20 h before addition of agonists.

### Immunoblot Analysis

Because N18TG2 cells can produce 2-arachidonoylglycerol (2-AG; Bisogno et al., 1997), cells at 90% confluency were serum-starved (20–24 h) and pretreated with the diacylglycerol lipase (DAGL) inhibitor THL (1 μM, 2 h) before stimulation with cannabinoid agonists. Following indicated drug treatments, cells were harvested with PBS-EDTA (2.7 mM KCl, 138 mM NaCl, 10.4 mM glucose, 1.5 mM KH_2_PO_4_, 8 mM Na_2_HPO_4_, 0.625 mM EDTA, pH 7.4). Cells were resuspended for 20 min on ice in an NP-40 lysis buffer that contained 10 mM NaHEPES, pH 7.9, 1.5 mM MgCl_2_, 10 mM KCl, 100 μM EDTA, 250 μM Na orthovanadate, 1 mM Na fluoride, 1% NP-40, 1 μM DTT, and a protease inhibitor cocktail (EMD Millipore) with broad specificity for the inhibition of aspartic, cysteine, and serine proteases as well as aminopeptidases. Lysates were clarified by centrifugation at 20,000 *g* at 4°C and supernatants were stored at −80°C. Protein concentrations were determined using the Bradford method with BSA as the standard (Bradford, 1976). Lysates were taken up in Laemmli’s sample buffer (62.5 mM Tris-HCl, pH 6.8, 2% SDS, 10% glycerol, 0.002% bromophenol blue, 100 mM DTT) and heated at 95°C for 5 min. Cell lysates were resolved by SDS-PAGE. Gels were pre-equilibrated in Towbin buffer (25 mM Tris Base, 192 mM glycine, 20% methanol, pH 8.3) for 30 min and proteins were transferred to nitrocellulose membranes using a BioRad Trans-Blot Cell. Blots were rinsed one time with Tris-buffered saline (TBS, 20 mM Tris-HCl, 137 mM NaCl, pH 7.4), blocked with Odyssey Blocking buffer, and then incubated with primary antibodies overnight at 4°C. Blots were washed four times with TBST (TBS containing 0.1% Tween-20), incubated with IRDye 800 CW goat anti-rabbit or IRDye 680 CW goat anti-mouse secondary antibodies (1:15,000) for 1 h at room temperature, followed by three washes with TBST and one wash with TBS. Immunoblots were imaged and bands were quantified by densitometry using Odyssey Infrared Imaging System software (LI-COR Biosciences, Lincoln, NE, USA).

### Fluorescence Microscopy Assay

N18TG2 cells were grown on glass coverslips. At 80% confluency, cells were treated with WIN55212-2 (1–1,000 nM, 20 min), latrunculin A (0.01 μg/ml, 30 min), or jasplakinolide (4 nM, 10 min). Following indicated drug treatments, cells were washed with PBS and fixed in 4% paraformaldehyde in cytoskeleton buffer (0.32 M sucrose, 10 mM (2-(4-morpholino)-ethane sulfonic acid, 3 mM MgCl_2_, 138 mM KCl, 2 mM EGTA, pH 6.1). Cells were then permeabilized with 0.5% Triton X-100, blocked with PBS/2% BSA, and treated with 0.1% sodium borohydride. Cells were washed in PBS, then stained with Alexa Fluor 488 phalloidin (200 units/ml) and Texas Red DNase I (9 μg/ml) to evaluate the relative amounts of F-actin and G-actin as previously described (Knowles and McCulloch, 1992). The fluorescence staining procedure was modified by using newer and brighter fluorochromes bound to phalloidin and DNAse I which label the F-actin and G-actin in cells, respectively. Following a wash with PBS, coverslips were mounted onto glass slides with Prolong Antifade reagent. Slides were viewed using a Nikon Eclipse E600 fluorescence microscope (Nikon Instruments, Inc., Melville, NY, USA) and images were digitalized using identical exposure time and brightness settings for all conditions. Quantification of fluorescence was performed using Image-Pro Plus 4.5 Software (Media Cybernetics, Inc., Rockville, MD, USA). Ratios of green (Alexa Fluor 488 phalloidin) to red (Texas Red DNase I) fluorescence were tabulated from three images of 15 or more cells per field. The excitation and emission wavelengths were: 495 nm/518 nm (Alexa Fluor 488 phalloidin) and 597 nm/615 nm (Texas Red DNase I).

### GRK2 RNA-Mediated Interference

N18TG2 cells were transfected with 100 nM GRK2-specific siRNA (mouse) or negative control siRNA-A using siRNA transfection reagent according to the manufacturer’s protocol (Santa Cruz Biotechnology). Negative control siRNA is a non-targeting siRNA that was used to verify the accuracy of GRK2-specific siRNA and was included in every siRNA experiment. In brief, N18TG2 cells (2 × 10^5^ cells/well) were plated on BD Falcon 6-well multiwell plates 24 h before transfection. Cells were then transfected with no siRNA (mock transfection), GRK2-specific siRNA (100 nM), or negative control siRNA (100 nM) for 6 h at 37°C, 5% CO_2_. Following transfections, cells were cultured in normal growth medium for 48 h and then serum-starved for 24 h before incubation with or without CB_1_ agonists. Following drug treatments, cells were harvested with PBS-EDTA, lysed, and protein modification was quantitated by immunoblotting experiments.

### Grb2 Co-immunoprecipitation With FAK

N18TG2 cells at 90% confluency were serum-starved (20–24 h) and pre-incubated with THL (1 μM, 2 h) before treatment with CB_1_ agonists. Following drug treatments, cells were harvested in PBS-EDTA, and pellets were lysed on ice with NP-40 lysis buffer plus protease inhibitor cocktail for 20 min. Lysates were clarified by centrifugation at 13,500 *g* at 4°C for 5 min and protein concentrations were determined (Bradford, 1976). Proteins (500 μg) were immunoprecipitated at 4°C with antibodies specific for FAK and collected with protein A/G PLUS-Agarose (Santa Cruz Biotechnology). The immune complexes were precipitated by centrifugation at 13,500 *g* for 5 min at 4°C, washed three times with ice-cold NP-40 buffer, and boiled in Laemmli’s sample buffer. Following centrifugation at 13,500 *g* for 5 min at 4°C, supernatants were collected and resolved by SDS-PAGE. Proteins were transferred onto nitrocellulose membranes and immunoblots were stained with anti-FAK or anti-Grb2 primary antibodies. Immunoblots were imaged and bands were quantified by densitometry using Odyssey Infrared Imaging System software.

### Statistical Analysis

Graphs and statistical analyses were generated using GraphPad Prism V software (La Jolla, CA, USA). The means between two independent samples were compared using the unpaired Student’s *t*-test. The one way ANOVA test followed by Dunnett’s multiple comparison posthoc test was used to compare the means between three or more independent samples.

## Results

### CB_1_ Stimulates Ras-Dependent Activation of the Raf/MEK/ERK Cascade in N18TG2 Cells

**Figure 1:**
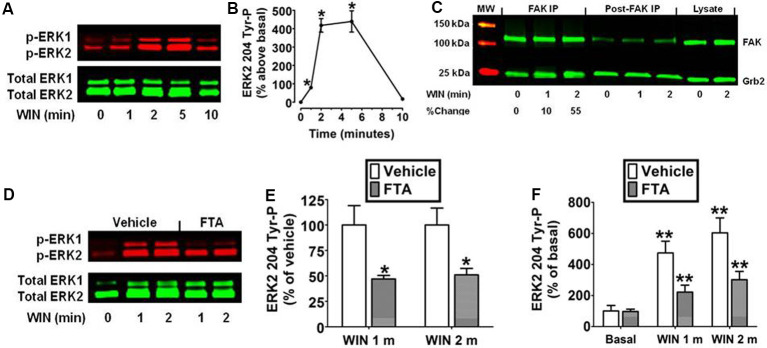
Cannabinoid receptors (CB_1_) stimulate Grb2/Sos1/Ras-dependent activation of extracellular signal-regulated kinase 2 (ERK2) phosphorylation at tyrosine 204 in N18TG2 cells. **(A,B)** Cells were treated with 0.01 μM WIN55212-2 (WIN) at 37°C for 1, 2, 5, and 10 min (m). Cell lysates were analyzed using western blots and representative blot images and analysis of ERK2 204 Tyr-P (normalized to total ERK2 levels) are shown. Data are reported as mean ± SEM of the % change over basal from three separate experiments. **(C)** Cells were treated with 0.01 μM WIN for 1 or 2 m at 37°C. Proteins from total cell lysate (500 μg) were immunoprecipitated with FAK antibodies and were immunoblotted with FAK and Grb2 antibodies. Data are the % change from basal Grb2 levels associated with FAK (normalized to total FAK at each time point). MW, molecular weight size marker. **(D–F)** Cells were pretreated for 15 m with a vehicle or the Ras inhibitor farnesylthiosalicylic acid (FTA, 10 μM) before treatment with 0.01 μM WIN for 1 or 2 m at 37°C. Cell lysates were analyzed using western blots and representative blot images and analysis of ERK2 204 Tyr-P (normalized to total ERK2 levels) are shown. Data are reported as mean ± SEM of panel **(E)** % of vehicle-treated ERK2 204 Tyr-P at the same time point or **(F)** the % of basal/time 0 ERK2 204 Tyr-P from three separate experiments. Significance was assessed using Student’s *t*-test [*p < 0.01 indicates significantly different from vehicle-treated (at the same time point); **p < 0.05 indicates significantly different from basal/time 0]. For each dataset, cells were cultured and experiments were completed on at least three separate occasions.

The N18TG2 neuroblastoma cell has been used as a model system to study signal transduction pathways regulated by the CB_1_ cannabinoid receptor because N18TG2 cells express mRNA and protein for CB_1_ but not CB_2_ receptors (Mukhopadhyay et al., 2006; Jones et al., 2008). Kinetic analysis revealed the synthetic CB_1_ agonist WIN55212-2 (0.01 μM) produced an increase in ERK2 204 Tyr-P in N18TG2 cells that reached maximal levels in 2–5 min and declined to baseline by 10 min (Figures 1A,B). ERK1 (phospho-p44 MAPK) and ERK2 (phospho-p42 MAPK) exhibited similar activation kinetics in N18TG2 cells (data not shown). Consequently, this report focused on the cellular mechanisms that control the level of ERK2 204 Tyr-P. The guanine nucleotide exchange factor Sos1 binds Grb2 at the plasma membrane where Sos1 activates Ras and the downstream Raf/MEK/ERK cascade (Roskoski, 2012). Phosphorylation of FAK at Tyr 925 promotes Grb2 binding to FAK and leads to FAK-mediated activation of the Ras-dependent MAPK pathway through its association with the Grb2/Sos1 complex (Schlaepfer et al., 1994; Schlaepfer and Hunter, 1996). Coimmunoprecipitation experiments confirmed a basal constitutive association of FAK with Grb2 in N18TG2 cells that increased following treatment of the cells with WIN55212-2 (0.01 μM; 1 min 9.8% ± 3.24 increase; 2 min 55.7% ± 17.38 increase; Figure 1C). To determine whether Ras signaling is necessary for ERK2 activation, we examined the effect of the Ras inhibitor FTA on WIN55212-2-stimulated ERK2 204 Tyr-P in N18TG2 cells (Haklai et al., 1998). As shown in Figures 1D–F, pretreatment of N18TG2 cells with FTA (10 μM) partially inhibited WIN55212-2-stimulated ERK2 204 Tyr-P (1 min 53.0% ± 3.40 inhibition; 2 min 49.0% ± 6.30 inhibition). Basal ERK2 204 Tyr-P was not altered by FTA or the vehicle for FTA (Figure 1F). These findings indicate CB_1_ activates the Grb2-Sos1-Ras axis to regulate ERK2 Tyr-P in N18TG2 cells.

### Inhibition of FAK 925 Tyr-P Abrogates CB_1_-Stimulated ERK2 204 Tyr-P in N18TG2 Cells

**Figure 2:**
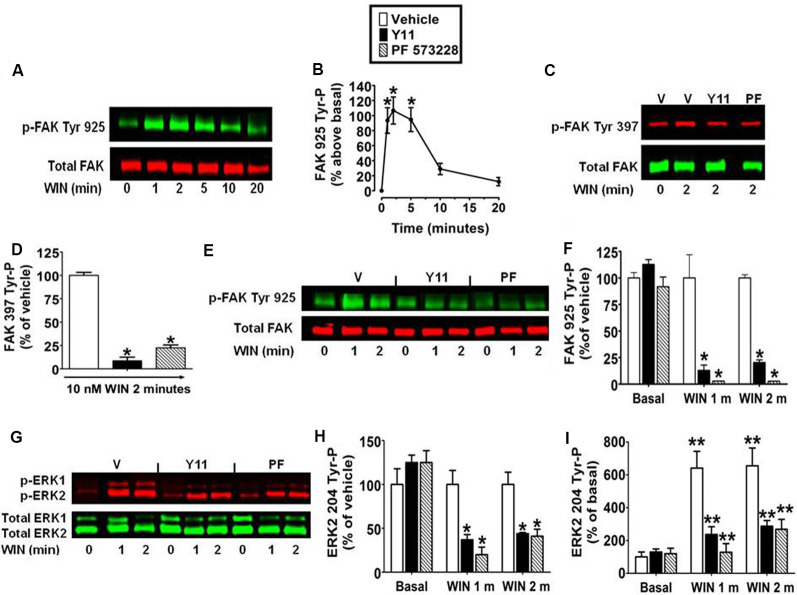
Inhibition of focal adhesion kinase (FAK) 925 Tyr-P abrogates CB_1_-stimulated ERK2 204 Tyr-P in N18TG2 cells. Cells were treated with 0.01 μM WIN55212-2 (WIN) at 37°C for 1, 2, 5, 10, or 20 min (m). **(A,B)** Cell lysates were analyzed using western blots and representative blot images and analysis of FAK 925 Tyr-P (normalized to total FAK levels) are shown. *p < 0.01 indicates significantly different from basal/time 0 using Student’s *t*-test. **(C–I)** Cells were pretreated for 15 m with 10 nM FAK inhibitor [Y11 or PF 573228 (PF)] before treatment with 0.01 μM WIN. Cell lysates were analyzed using western blots and representative blot images and analysis of **(C,D)** FAK 397 Tyr-P, **(E,F)** FAK 925 Tyr-P, and **(G–I)** ERK2 204 Tyr-P (normalized to total FAK or ERK2 levels) are shown. Data are reported as mean ± SEM of **(B)** the % change over basal FAK 925 Tyr-P, **(D,F,H)** the % of vehicle-treated FAK 397, FAK 925 and ERK2 204 Tyr-P at the same time point, and **(I)** the % of basal/time 0 from three separate experiments. For 2C-I, significance was assessed using One Way ANOVA followed by Dunnett’s multiple comparisons posthoc test [*p < 0.01 indicates significantly different from vehicle-treated (at the same time point); **p < 0.05 indicates significantly different from basal/time 0]. For each dataset, cells were cultured and experiments were completed on at least three separate occasions.

The kinetic analysis also revealed WIN55212-2 (0.01 μM) produced an increase in FAK 925 Tyr-P that reached maximal levels in 1–5 min and declined to near basal levels by 20 min (Figures 2A,B). FAK activation is initiated by FAK 397 Tyr-P. Phosphorylated FAK Tyr 397 binds Src which can then phosphorylate FAK on additional Tyr residues including Tyr 925 (Schlaepfer and Hunter, 1996). N18TG2 cells were pretreated with the FAK inhibitors PF 573228 and Y11 that block FAK 397 Tyr-P (Schlaepfer and Hunter, 1996; Slack-Davis et al., 2007; Golubovskaya et al., 2012). PF 573228 (10 nM, 77.5% ± 3.15 inhibition) and Y11 (10 nM, 91.2% ± 3.75 inhibition) significantly reduced WIN55212-2-stimulated FAK 397 Tyr-P (Figures 2C,D). WIN55212-2-stimulated FAK 925 Tyr-P was also significantly reduced by Y11 (10 nM, 1 min 87.1% ± 5.08 inhibition; 2 min 79.6% ± 2.40 inhibition) and PF 573228 (10 nM, 1 min 97.2% ± 0.07 inhibition; 2 min 97.3% ± 0.08 inhibition) which suggests CB_1_-stimulated FAK 925 Tyr-P requires FAK 397 Tyr-P in N18TG2 cells (Figures 2E,F). Studies in other experimental systems have demonstrated that FAK 925 Tyr-P can promote FAK-mediated activation of MAPK (Schlaepfer et al., 1994; Schlaepfer and Hunter, 1996; Mitra et al., 2006; Cheung et al., 2008). In N18TG2 cells, WIN55212-2 stimulated ERK2 204 Tyr-P was partially inhibited by Y11 (1 min 63.0% ± 5.98 inhibition; 2 min 56.0% ± 8.52 inhibition) and PF 573228 (1 min 80.0% ± 8.5 inhibition; 2 min 59.0% ± 8.94 inhibition) suggesting FAK 925 Tyr-P is regulatory for CB_1_-stimulated ERK2 204 Tyr-P (Figures 2G–I).

### CB_1_-Stimulated FAK 925 Tyr-P and ERK2 204 Tyr-P Requires an Intact Actin Cytoskeleton in N18TG2 Cells

**Figure 3:**
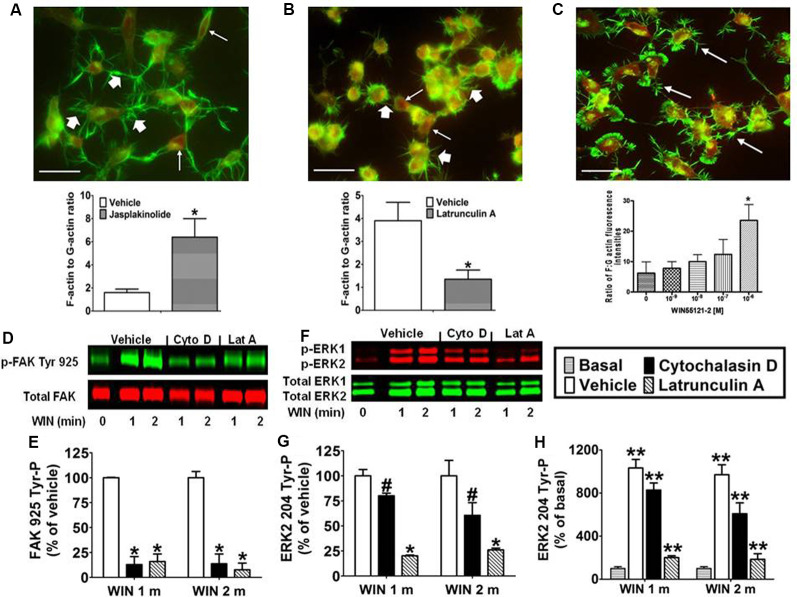
CB_1_-stimulated FAK phosphorylation at tyrosine 925 and ERK2 phosphorylation at tyrosine 204 requires an intact actin cytoskeleton in N18TG2 cells. **(A–C)** Cells grown on glass coverslips were treated with jasplakinolide (4 nM, 10 min), latrunculin A (0.01 μg/ml, 30 min), or WIN55212-2 (1–1,000 nM, 20 min). Actin remodeling was detected by fluorescent double labeling of F-actin (Alexa Fluor 488 phalloidin, short white arrow) and G-actin [Texas Red DNase I, long white arrow; neurite outgrowths, white arrow in panel **(C)**]. F-actin to G-actin ratios were quantified from green and red staining intensities using Image Pro 4.5 software. *p < 0.01 indicates significantly different from vehicle-treated using Student’s *t*-test. **(D–H)** Cells were pretreated with cytochalasin D (2 μM, Cyto D) or latrunculin A (1 μM, Lat A) before treatment with 0.01 μM WIN55212-2 (WIN) for 1 or 2 min (m) at 37°C. Cell lysates were analyzed using western blots and representative blot images and analysis of FAK 925 Tyr-P (normalized to total FAK levels) and ERK2 204 Tyr-P (normalized to total ERK2 levels) are shown. Data are reported as mean ± SEM of **(E)** % of vehicle-treated FAK 925 Tyr-P at the same time point, **(G)** % of vehicle-treated ERK2 204 Tyr-P at the same time point, and **(H)** % of basal/time 0 ERK2 204 Tyr-P from three separate experiments. Significance was assessed using One Way ANOVA followed by Dunnett’s multiple comparisons posthoc test [^#^*p* < 0.05, *p < 0.01 indicates significantly different from vehicle-treated (at the same time point); **p < 0.05 indicates significantly different from basal/time 0]. For each dataset in panels **(D–H)**, cells were cultured and experiments were completed on at least three separate occasions.

Integrins are cell adhesion molecules that link the actin cytoskeleton to proteins of the ECM and form the architectural backbone of focal adhesions. Focal adhesions are present in N18TG2 cells as confirmed by anti-vinculin staining, and localization of FAK phosphorylated at Tyr 925 in response to CB_1_ stimulation in these structures (unpublished data from our laboratory). Actin exists as a dynamic equilibrium mixture of two forms: polymeric filamentous actin (F-actin) and monomeric globular actin (G-actin; Coumans et al., 1997). The F-actin to G-actin ratio serves as a marker of actin cytoskeleton homeostasis. Studies were conducted to confirm that remodeling of the actin cytoskeleton could be induced in N18TG2 cells using jasplakinolide and latrunculin A. Jasplakinolide is a potent inducer of actin polymerization by stimulating actin filament nucleation, while latrunculin A disrupts the actin cytoskeleton by binding monomeric G-actin and preventing actin polymerization (Spector et al., 1983; Coué et al., 1987; Allingham et al., 2006). In N18TG2 cells, jasplakinolide (4 nM) increased the F-actin to G-actin ratio suggesting actin polymerization, while latrunculin A (0.01 μg/ml) diminished the F-actin to G-actin ratio suggesting actin depolymerization (Figures 3A,B). Cells treated with jasplakinolide also had numerous prominent neurites, while latrunculin A-treated cells had few small processes (Figures 3A,B). WIN55212-2 increased the F-actin to G-actin ratio in a dose-dependent manner in N18TG2 cells (Figure 3C).

Given the important role that the actin cytoskeleton plays in focal adhesions, we investigated the effect of chemical disruption of the actin cytoskeleton on CB_1_-stimulated FAK 925 Tyr-P and ERK2 204 Tyr-P. To accomplish this, N18TG2 cells were pretreated with the actin disruptors (latrunculin A, cytochalasin D) at concentrations that were based on determined IC_50_ values (Spector et al., 1983; Goddette and Frieden, 1986; Coué et al., 1987; Sampath and Pollard, 1991). Cytochalasin D binds directly to the growing ends of actin filaments to block assembly and disassembly of individual actin monomers from the bound end (Goddette and Frieden, 1986; Sampath and Pollard, 1991). In the present study, cytochalasin D (2 μM) reduced WIN55212-2-stimulated FAK 925 Tyr-P (1 min 87.1% ± 8.01 inhibition; 2 min 86.3% ± 9.80 inhibition; Figures 3D,E). Similarly, latrunculin A (1 μM) inhibited WIN55212-2-stimulated FAK 925 Tyr-P (1 min 84.0% ± 7.48 inhibition; 2 min 92.4% ± 6.75 inhibition; Figures 3D,E). Cytochalasin D (1 min 19.9% ± 2.04 inhibition; 2 min 39.4% ± 12.63 inhibition) and latrunculin A (1 min 79.8% ± 0.71 inhibition; 2 min 73.9% ± 1.81 inhibition) also reduced ERK2 204 Tyr-P (Figures 3F–H). Basal FAK 925 Tyr-P and ERK2 204 Tyr-P were not altered by either inhibitor or the vehicles for these inhibitors (data not shown).

### CB_1_-Stimulated FAK 925 Tyr-P and ERK2 204 Tyr-P Are Mediated by Integrins and Receptor Tyrosine Kinases in N18TG2 Cells

**Figure 4:**
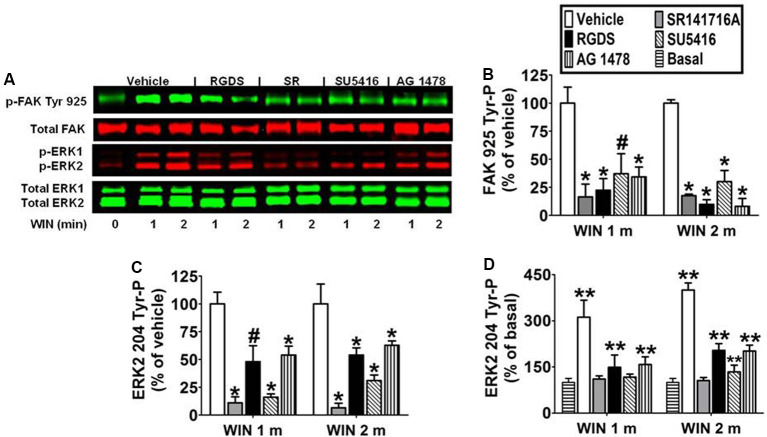
CB_1_-stimulated FAK phosphorylation at tyrosine 925 and ERK2 phosphorylation at tyrosine 204 are mediated by integrins and receptor tyrosine kinases in N18TG2 cells. **(A–D)** Cells were pretreated for 15 min (m) with the integrin antagonist RGDS peptide (100 μM), CB_1_ antagonist SR141716A (1 μM, SR), Flk-1 VEGFR antagonist SU 5416 (1 μM), or EGFR antagonist AG 1478 (2 μM) before treatment with 0.01 μM WIN55212-2 (WIN) for 1 or 2 m at 37°C. Cell lysates were analyzed using western blots and representative blot images are shown. Data are reported as mean ± SEM of **(B)** % of vehicle-treated FAK 925 Tyr-P (normalized to total FAK levels) at the same time point, **(C)** % of vehicle-treated ERK2 204 Tyr-P (normalized to total ERK2 levels) at the same time point, and **(D)** % of basal/time 0 ERK2 204 Tyr-P (normalized to total ERK2 levels). Significance was assessed using One Way ANOVA followed by Dunnett’s multiple comparisons posthoc test [^#^*p* < 0.05, *p < 0.01 indicates significantly different from vehicle-treated (at the same time point); **p < 0.05 indicates significantly different from basal/time 0]. For each dataset, cells were cultured and experiments were completed on at least three separate occasions.

Immunoblotting analysis revealed the CB_1_ antagonist SR141716A (1 μM) blocked WIN55212-2-stimulated FAK 925 Tyr-P (1 min 83.5% ± 11.40 inhibition; 2 min 82.4% ± 1.34 inhibition) and ERK2 204 Tyr-P (1 min 89.8% ± 5.39 inhibition; 2 min 93.4% ± 4.07 inhibition) which indicates these are CB_1_-dependent events in N18TG2 cells (Figures 4A–D). Previous studies in our laboratory have demonstrated that CB_1_ engages in cross-talk with fibronectin-binding integrins, as well as Flk-1 VEGFRs and EGFRs to stimulate maximal FAK activation in N18TG2 cells (Dalton et al., 2013). In data not shown, CB_1_ failed to stimulate FAK 925 Tyr-P in the absence of integrin activation in suspended N18TG2 cells, while ERK2 204 Tyr-P was reduced by approximately 50%. These findings led us to hypothesize that both FAK 925 Tyr-P and ERK2 Tyr-P are adhesion-dependent and involve cross-talk between CB_1_ and integrins.

To determine if CB_1_-stimulated FAK 925 Tyr-P and ERK2 204 Tyr-P are mediated by integrin receptors, N18TG2 cells were pretreated with the integrin antagonist RGDS peptide (100 μM) at concentrations that maximally inhibited CB_1_-stimulated maximal FAK activation (Dalton et al., 2013). ECM proteins, such as fibronectin, possess an RGD (Arg-Gly-Asp) amino acid sequence that is recognized by RGD-binding integrins. Integrin binding peptides containing the RGD sequence compete with ECM proteins for binding to these integrins and subsequently block processes mediated by RGD-binding integrins (Matsuno et al., 1994). In the present study, RGDS peptide blocked WIN55212-2-stimulated FAK 925 Tyr-P (1 min 77.5% ± 10.39 inhibition; 2 min 90.2% ± 4.20 inhibition), and produced a less robust reduction in WIN55212-2-stimulated ERK2 204 Tyr-P (1 min 48.5% ± 14.43 inhibition; 2 min 54.8% ± 6.41 inhibition; Figures 4A–D). Finally, RGDS peptide did not affect basal FAK 925 Tyr-P or ERK2 204 Tyr-P under these experimental conditions (data not shown).

To determine if CB_1_ transactivates Flk-1 VEGFRs and EGFRs to regulate FAK 925 Tyr-P and ERK2 204 Tyr-P, N18TG2 cells were pretreated with selective Flk-1 VEGFR and EGFR inhibitors at concentrations that maximally inhibited CB_1_-stimulated maximal FAK activation (Dalton et al., 2013). The Flk-1 VEGFR antagonist SU 5416 (1 μM) inhibited CB_1_-stimulated FAK 925 Tyr-P (1 min 62.8% ± 17.78 inhibition; 2 min 69.9% ± 9.96 inhibition), as did the EGFR antagonist AG 1478 (2 μM; 1 min 65.6% ± 8.66 inhibition; 2 min 91.9% ± 6.99 inhibition; Figures 4A,B). SU 5416 (1 min 82.1% ± 1.26 inhibition; 2 min 69.0% ± 5.00 inhibition) and AG 1478 (1 min 46.8% ± 8.62 inhibition; 2 min 37.2% ± 4.00 inhibition) also blocked ERK2 204 Tyr-P in N18TG2 cells (Figures 4A,C,D). Basal FAK 925 Tyr-P and ERK2 204 Tyr-P were not changed by either RTK inhibitor or the DMSO vehicle for these inhibitors (data not shown).

### CB_1_-Stimulated FAK 925 Tyr-P and ERK2 204 Tyr-P Are Mediated by Gβγ and GRK2 in N18TG2 Cells

**Figure 5:**
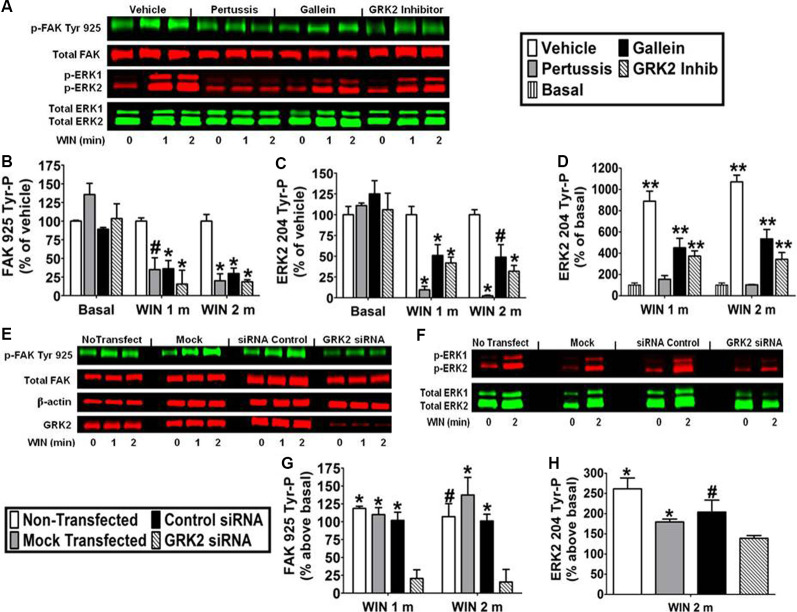
CB_1_-stimulated FAK phosphorylation at tyrosine 925 and ERK2 phosphorylation at tyrosine 204 are mediated by Gβγ and GRK2 in N18TG2 cells. **(A–D)** Cells were pretreated with the G_i/o_ inhibitor pertussis toxin (100 ng/mL, 20 h), Gβγ inhibitor gallein (10 μM, 15 min), or GRK2 inhibitor (1 μM, 15 min) before treatment with 0.01 μM WIN55212-2 (WIN) for 1 or 2 min (m) at 37°C. Cell lysates were analyzed using western blots and representative blot images are shown. Data are reported as mean ± SEM of **(B)** % of vehicle-treated FAK 925 Tyr-P (normalized to total FAK levels) at the same time point, **(C)** % of vehicle-treated ERK2 204 Tyr-P (normalized to total ERK2 levels) at the same time point, and **(D)** % of basal/time 0 ERK2 204 Tyr-P (normalized to total ERK2 levels). Significance was assessed using One Way ANOVA followed by Dunnett’s multiple comparisons posthoc test [*p < 0.01, ^#^*p* < 0.05 indicates significantly different from vehicle-treated (at the same time point); **p < 0.05 indicates significantly different from basal/time 0]. **(E–H)** Cells (2 × 10^5^) were transfected with no siRNA (mock transfection), GRK2-specific siRNA (100 nM), or negative control siRNA (100 nM) before treatment with 0.01 μM WIN for 2 min at 37°C. Immunoblot analysis was performed and data are reported as mean ± SEM of the % change over basal **(G)** FAK 925 Tyr-P (normalized to total FAK levels) and **(H)** ERK2 204 Tyr-P (normalized to total ERK2 levels). Significance was assessed using One Way ANOVA followed by Dunnett’s multiple comparisons posthoc test (*p < 0.01, ^#^*p* < 0.05 indicates significantly different from GRK2 siRNA at the same time point). For each dataset, cells were cultured and experiments were completed on at least three separate occasions.

Pretreatment with the G_i/o_ inhibitor pertussis toxin (100 ng/ml) decreased the effect of WIN55212-2 (0.01 μM) on FAK 925 Tyr-P (1 min 65.1% ± 15.94 inhibition; 2 min 80.0% ± 9.32 inhibition) and ERK2 204 Tyr-P (1 min 90.4% ± 4.38 inhibition; 2 min 97.5% ± 0.76 inhibition) indicating the requirement for CB_1_ stimulation of G_i/o_ proteins in N18TG2 cells (Figures 5A–D). The signaling, trafficking, and degradation of many GPCRs are regulated by GPCR-desensitizing GRKs and β-arrestins which impair GPCR-mediated signaling events (Pitcher et al., 1998). GRKs, such as GRK2 and GRK3, as well as β-arrestins regulate CB_1_ sensitivity which led us to investigate the involvement of GRK2 in this process in N18TG2 cells (Jin et al., 1999; Kouznetsova et al., 2002; Breivogel et al., 2008). Dose-response studies conducted in our laboratory determined that a small molecule GRK2 inhibitor (Iino et al., 2002) reduced WIN55212-2-stimulated FAK 925 Tyr-P in N18TG2 cells maximally at 1 μM (data not shown). In the current study, the GRK2 inhibitor (1 μM) significantly reduced WIN55212-2-stimulated FAK 925 Tyr-P (1 min 84.4% ± 18.52 inhibition; 2 min 81.4% ± 2.76 inhibition), as well as ERK2 204 Tyr-P (1 min 62.9% ± 7.94 inhibition; 2 min 67.5% ± 5.86 inhibition; Figures 5A–D). To confirm the contribution of GRK2 to CB_1_-stimulated FAK 925 Tyr-P and ERK2 204 Tyr-P, N18TG2 cells were transfected with GRK2-specific siRNA. Immunoblotting analysis demonstrated that GRK2 expression was significantly reduced in N18TG2 cells by GRK2-specific siRNA when compared with non-transfected cells, mock transfected (no siRNA) cells, or cells transfected with negative control siRNA (Figure 5E). Transfection with GRK2-specific siRNA inhibited WIN55212-2-stimulated FAK 925 Tyr-P and ERK2 204 Tyr-P without influencing total FAK or β-actin levels in these cells (Figures 5E–H). These data confirm that GRK2 mediates CB_1_-stimulated FAK 925 Tyr-P and ERK2 204 Tyr-P in N18TG2 cells. Like GRK2, studies have demonstrated that Gβγ plays a critical role in GPCR signaling, while FAK and ERK1/2 have emerged as key downstream effectors of Gβγ (Knezevic et al., 2009; Dalton and Howlett, 2012). To investigate the involvement of Gβγ in CB_1_-stimulated FAK 925 Tyr-P and ERK2 204 Tyr-P, N18TG2 cells were pretreated with the Gβγ inhibitor gallein (10 μM) at concentrations that block CB_1_-stimulated ERK1/2 activation (Lehmann et al., 2008; Dalton and Howlett, 2012). Gallein reduced WIN55212-2-stimulated FAK 925 Tyr-P (1 min 63.8% ± 10.97 inhibition; 2 min 70.3% ± 7.27 inhibition) and ERK2 204 Tyr-P (1 min 43.1% ± 3.51 inhibition; 2 min 45.9% ± 15.06 inhibition; Figures 5A–D). Finally, basal FAK 925 Tyr-P and ERK2 204 Tyr-P were not altered by these inhibitors or their vehicles (Figures 5A–D).

## Discussion

In N18TG2 neuronal cells, FAK activation begins with FAK 397 Tyr-P, which generates a binding site for Src, resulting in Src activation and Src subsequent phosphorylation of FAK on Tyr 925 (Schlaepfer et al., 1994; Schlaepfer and Hunter, 1996; Parsons, 2003). FAK 925 Tyr-P creates a Src-homology-2-binding site for the adaptor protein Grb2 which links FAK to Grb2-Sos1-mediated activation of the Ras/Raf/MEK/ERK1/2 cascade (Schlaepfer et al., 1994; Schlaepfer and Hunter, 1996; Mitra et al., 2006; Cheung et al., 2008; Roskoski, 2012). CB_1_-stimulated FAK 925 Tyr-P was rapid (0–5 min) and transient, with a more prolonged decline (5–20 min). Like FAK, ERK2 activation is regulated by reversible protein phosphorylation and is dependent on Tyr 204 phosphorylation in the ERK2 activation loop with similar activation kinetics to those of FAK (Roskoski, 2012). The FAK inhibitors Y11 and PF 573228 abolished CB_1_-stimulated FAK 925 Tyr-P, and partially blocked CB_1_-stimulated ERK2 204 Tyr-P. We propose that FAK 925 Tyr-P is a factor in CB_1_-stimulated ERK2 activation in N18TG2 cells. Evidence supporting this includes the Src-dependence of CB_1_-stimulated FAK 925 Tyr-P coupled with our previous demonstration that the Src inhibitor PP2 (2 μM) reduces CB_1_-stimulated ERK1/2 204 Tyr-P by approximately 50% in N18TG2 cells (Dalton and Howlett, 2012), similar in magnitude to ERK2 inhibition produced by the FAK inhibitors Y11 and PF 573228 in this study. Our findings also confirm the observation that FAK 925 Tyr-P creates a binding site for Grb2 (Schlaepfer et al., 1994; Schlaepfer and Hunter, 1996; Mitra et al., 2006). In N18TG2 cells, FAK Tyr 925 was phosphorylated and associated with Grb2 under serum-starved basal conditions. CB_1_ agonists promoted an increase in FAK-Grb2 association. Grb2 forms a complex with the Ras activator Sos1 which links FAK to activation of the Ras/Raf/MEK/ERK1/2 cascade (Schlaepfer et al., 1994; Schlaepfer and Hunter, 1996; Mitra et al., 2006; Roskoski, 2012). Taken together, these results imply that a component of CB_1_-stimulated ERK2 204 Tyr-P requires FAK 397 Tyr-P, Src activation, and FAK 925 Tyr-P in N18TG2 cells.

The murine neuroblastoma N18TG2 cell is a cell line of neuronal origin. Previous studies have demonstrated that the neuronal isoform of FAK, FAK+6,7, is the major FAK isoform found in hippocampal slices but not astrocytes or microglia (Derkinderen et al., 1996, 2001). The endocannabinoids anandamide and 2-AG both stimulated FAK+6,7 397 Tyr-P in hippocampal slices that was followed by FAK+6,7 925 Tyr-P ((Derkinderen et al., 2001). Moreover, endocannabinoid-stimulated FAK+6,7 925 Tyr-P was dependent specifically on the Src kinase Fyn in hippocampal slices (Derkinderen et al., 2001). Our work in N18TG2 cells is consistent with what has been shown in hippocampal slices regarding how CB_1_ stimulates FAK 925 Tyr-P. We have shown FAK 397 Tyr-P precedes FAK 925 Tyr-P, while work in our lab has also confirmed that FAK 925 Tyr-P is Src-dependent in N18TG2 cells. At present, we do not know the specific FAK isoform expressed in N18TG2 cells and we have not confirmed that FAK 925 Tyr-P is mediated specifically by Fyn. Thus, it is possible the FAK-dependent mechanism linking CB_1_ to ERK activation is different in N18TG2 cells compared to hippocampal slices and involves a different FAK isoform and a different Src family member. Although our pathway leading from CB_1_ to FAK 925 Tyr-P in the cloned N18TG2 neuronal cell line is similar to what has been observed in hippocampal slices, future studies would need to be done in hippocampal slices to examine the effects of FAK inhibition on CB_1_-stimulated ERK activation to determine if the mechanism is similar to what we report herein.

Studies suggest that actin stress fibers play a significant role in protein recruitment to focal adhesions (Oakes et al., 2012). For this reason, the Tyr-P and activation of FAK are critically dependent on the integrity of the actin cytoskeleton. Disruption of the actin cytoskeleton has been shown to inhibit focal adhesion formation, block Src and FAK translocation to focal adhesions, and block FAK Tyr-P (Lipfert et al., 1992; Fincham et al., 1996; Chen et al., 2000). We confirmed herein that the integrity of the actin cytoskeleton plays an important role in CB_1_-stimulated FAK 925 Tyr-P and ERK2 204 Tyr-P in N18TG2 cells, as treatment with the actin-disrupting drugs cytochalasin D and latrunculin A blocked these events.

FAK is a Tyr kinase that localizes to focal adhesions in which integrins link the actin cytoskeleton to proteins of the ECM. Focal adhesion signaling is mediated by integrins and we have demonstrated that fibronectin (α5β1) and laminin (α6β1, α7β1) integrin receptors are present in N18TG2 cells (Dalton et al., 2013). Integrin activation involves a combination of integrin clustering, as well as ligand occupancy/activation of integrins which mediates cell adhesion to the ECM (Parsons, 2003). α5β1 fibronectin receptors bind to an RGD sequence located in the ECM protein fibronectin, and synthetic peptides, such as RGDS, are effective RGD-binding integrin antagonists because they occupy these integrin sites (Matsuno et al., 1994). Our demonstration that the RGDS peptide significantly reduced both FAK 925 Tyr-P and ERK2 204 Tyr-P in adherent N18TG2 cells attached to their own ECM supports the conclusion that CB_1_ engages in inter-receptor cross-talk with integrins, as observed for CB_1_ in the brain and several other GPCRs (Slack, 1998; Karanian et al., 2005; Teoh et al., 2012; Wang et al., 2018). Furthermore, studies in hippocampal slice cultures demonstrated an integrin antagonist blocks CB_1_-stimulated FAK and ERK activation (Karanian et al., 2005). In our previous work we demonstrated that CB_1_ transactivates multiple integrins to stimulate maximal FAK activation in N18TG2 cells (Dalton et al., 2013) but whether GPCRs stimulate integrin activation directly by Gα subunits remains a subject of investigation (Gong et al., 2010).

FAK is an important signaling effector for RTKs and Flk-1 VEGFR and EGFR ligands stimulated FAK Tyr-P (Abedi and Zachary, 1997; Rousseau et al., 2000; Long et al., 2010). We confirmed previous studies that indicated CB_1_ transactivates Flk-1 VEGFRs and EGFRs to stimulate ERK1/2 activation (Dalton and Howlett, 2012) and maximal FAK catalytic activity in N18TG2 cells (Dalton et al., 2013), as we demonstrated that inhibition of Flk-1 VEGFRs and EGFRs reduced both. Our data suggest that cooperative signaling between integrins and these RTKs may be involved in CB_1_-stimulated FAK 925 Tyr-P, which would add another interesting dimension to how CB_1_ regulates mitogenic signaling in neuronal cells. However, integrins and RTKs can also stimulate Ras-mediated activation of the MAPK cascade independently of FAK and do so by binding Grb2-Sos1 directly or indirectly *via* a Shc protein (van der Geer and Pawson, 1995; Rojas et al., 1996; Wary et al., 1996; Kroll and Waltenberger, 1997; McKay and Morrison, 2007). Based on the partial dependence of CB_1_-stimulated ERK2 204 Tyr-P on FAK 925 Tyr-P, it is clear that a component of CB_1_-mediated ERK2 signaling is accomplished autonomously from FAK in N18TG2 cells.

Inhibition of FAK 925 Tyr-P and ERK2 204 Tyr-P with pertussis toxin confirmed the involvement of G_i/o_-coupled CB_1_ receptors in these processes, and further evidence indicated that this is mediated by G_i/o_βγ subunits in N18TG2 cells (Dalton and Howlett, 2012). The Gβγ inhibitor gallein significantly reduced FAK 925 Tyr-P and ERK2 204 Tyr-P demonstrating that FAK and ERK are Gβγ effectors in N18TG2 cells. In addition to Gβγ, GPCRs are regulated by GRKs which participate with β-arrestins in the desensitization-dependent phosphorylation and transient internalization of GPCRs to terminate GPCR-mediated responses (Pitcher et al., 1998; Jin et al., 1999; Kouznetsova et al., 2002; Breivogel et al., 2008). GRK2 is a Gβγ-dependent kinase that has been shown to modulate CB_1_-mediated signaling events (Kouznetsova et al., 2002). We found that a GRK2 inhibitor as well as siRNA silencing of GRK2 expression reduced FAK 925 Tyr-P and ERK2 204 Tyr-P. Information has now emerged that GRK2 can trigger signal propagation from GPCRs and participate in cross-talk between integrins and sphingosine-1-phosphate receptors (Penela et al., 2008). These studies propose that GRK2 forms a complex with G_i/o_βγ at the plasma membrane where it acts as a scaffold to recruit signaling proteins, such as GIT1, that mediate signals emanating from integrin/sphingosine-1-phosphate stimulation to ultimately promote ERK1/2 activation, focal adhesion turnover, modification of focal adhesion-associated proteins, and cell migration (Penela et al., 2008). Based on this model and our findings with gallein, we speculate that G_i/o_βγ-bound GRK2 acts in a kinase-independent manner to mediate CB_1_-stimulated FAK and ERK2 activation in N18TG2 cells. Future research is needed to understand the roles GRK2 and G_i/o_βγ play in this process. It will also be interesting to determine if other GRKs that have been implicated in CB_1_ desensitization behave like GRK2 or if these GRKs mediate the decline in CB_1_-stimulated FAK and ERK2 activation in N18TG2 cells (Jin et al., 1999). Recent evidence that GRK5 is involved in CB_2_-induced upregulation and increased activity of 5-HT_2A_ receptors in neuronal cells also invites opportunities for further investigation (Franklin and Carrasco, 2013).

## Conclusion

In summary, our data identify novel functions for FAK in CB_1_-stimulated MAPK signaling and characterize an actin cytoskeletal and G_i/o_βγ-GRK2-mediated signaling pathway utilized by CB_1_ to stimulate FAK-mediated MAPK activation. These results provide a mechanism where FAK and MAPK integrate signals from CB_1_, integrins, and RTKs. CB_1_ signaling through FAK and MAPK may play important physiological roles in CB_1_ regulation of the actin cytoskeleton, neuronal migration, proliferation, and generation of neuritic processes (Harkany et al., 2008; Zorina et al., 2010; Jung et al., 2011; Peng and Guan, 2011; Gaffuri et al., 2012; Roskoski, 2012; Shum et al., 2020).

## Data Availability Statement

The raw data supporting the conclusions of this article will be made available by the authors, without undue reservation.

## Author Contributions

GD, SC, JM, DN, and AH conceived and designed the experiments, analyzed the data, and drafted and finalized the manuscript.

## Conflict of Interest

The authors declare that the research was conducted in the absence of any commercial or financial relationships that could be construed as a potential conflict of interest.
